# An exploratory investigation on spatiotemporal parameters, margins of stability, and their interaction in bilateral vestibulopathy

**DOI:** 10.1038/s41598-021-85870-7

**Published:** 2021-03-19

**Authors:** Nolan Herssens, Wim Saeys, Luc Vereeck, Kenneth Meijer, Raymond van de Berg, Vincent Van Rompaey, Christopher McCrum, Ann Hallemans

**Affiliations:** 1 Department of Rehabilitation Sciences, Ghent University, Campus UZ Gent, Corneel Heymanslaan 10, Building B3, 9000 Ghent, Belgium; 2 Department of Rehabilitation Sciences and Physiotherapy/Movant, Faculty of Medicine and Health Sciences, University of Antwerp, Antwerp, Belgium; 3 Multidisciplinary Motor Centre Antwerp (M2OCEAN), University of Antwerp, Antwerp, Belgium; 4 RevArte Rehabilitation Hospital, Edegem, Belgium; 5 Department of Nutrition and Movement Sciences, NUTRIM School of Nutrition and Translational Research in Metabolism, Maastricht University Medical Centre+, Maastricht, The Netherlands; 6 Division of Balance Disorders, Department of Otorhinolaryngology and Head and Neck Surgery, Faculty of Health Medicine and Life Sciences, School for Mental Health and Neuroscience, Maastricht University Medical Centre+, Maastricht, The Netherlands; 7 Faculty of Physics, Tomsk State University, Tomsk, Russia; 8 Department of Otorhinolaryngology and Head and Neck Surgery, Antwerp University Hospital, Edegem, Belgium; 9 Faculty of Medicine and Health Sciences, University of Antwerp, Antwerp, Belgium

**Keywords:** Movement disorders, Peripheral neuropathies

## Abstract

Integration of accurate vestibular, visual, and proprioceptive information is crucial in managing the centre of mass in relation to the base of support during gait. Therefore, bilateral loss of peripheral vestibular function can be highly debilitating when performing activities of daily life. To further investigate the influence of an impaired peripheral vestibular system on gait stability, spatiotemporal parameters, step-to-step variability, and mechanical stability parameters were examined in 20 patients with bilateral vestibulopathy and 20 matched healthy controls during preferred overground walking. Additionally, using a partial least squares analysis the relationship between spatiotemporal parameters of gait and the margins of stability was explored in both groups. Patients with bilateral vestibulopathy showed an increased cadence compared to healthy controls (121 ± 9 vs 115 ± 8 steps/min; p = 0.02; *d* = 0.77). In addition, although not significant (p = 0.07), a moderate effect size (*d* = 0.60) was found for step width variability (Coefficient of Variation (%); Bilateral vestibulopathy: 19 ± 11%; Healthy controls: 13 ± 5%). Results of the partial least squares analysis suggest that patients with peripheral vestibular failure implement a different balance control strategy. Instead of altering the step parameters, as is the case in healthy controls, they use the single and double support phases to control the state of the centre of mass to improve the mechanical stability.

## Introduction

In patients with bilateral vestibulopathy (BVP), a bilaterally absent or reduced function of the peripheral vestibular organs, vestibular nerves or both, is present^1,2^. Symptoms reported by almost all patients include movement-induced blurred vision during walking or quick head movements (i.e., oscillopsia) and unsteadiness during walking and standing that worsens in darkness and on uneven terrain^3^. Additionally, a wide variety of symptoms has been reported in literature, discussed in a recent review by Lucieer, et al.^3^, including but not limited to: hearing loss^2,4^, psychological symptoms^5,6^, spatial and non-spatial cognitive deficits^7,8^ and impaired quality of life^4,6^. Consequently, the symptoms of oscillopsia and postural imbalance are included in the diagnostic criteria for bilateral vestibulopathy published by the Classification Committee of the Bárány Society^9^. Both these symptoms can be directly related to the impaired function of the vestibular system, specifically the vestibulo-ocular reflex (VOR) and the vestibulo-spinal reflex (VSR), respectively^9^.

An impaired VSR, in particular, is highly debilitating when performing daily life activities, as adequate balance control is required in both static and dynamic situations^10^. Achieving, maintaining and restoring balance control depends on the multisensory integration of vestibular, visual and proprioceptive information^10^. The loss of vestibular input interferes with this complex process in BVP-patients. Both during quiet standing and dynamic situations, as well as during internally and externally triggered perturbations to balance, the centre of mass (CoM) should be controlled in relation to the base of support (BoS)^11^. The relationship between the CoM and the BoS, while different during quiet standing and dynamic situations such as walking, is continuously monitored and maintained in such a way that prevents the CoM of reaching a point that cannot be recovered from^12^.

**Figure 1:**
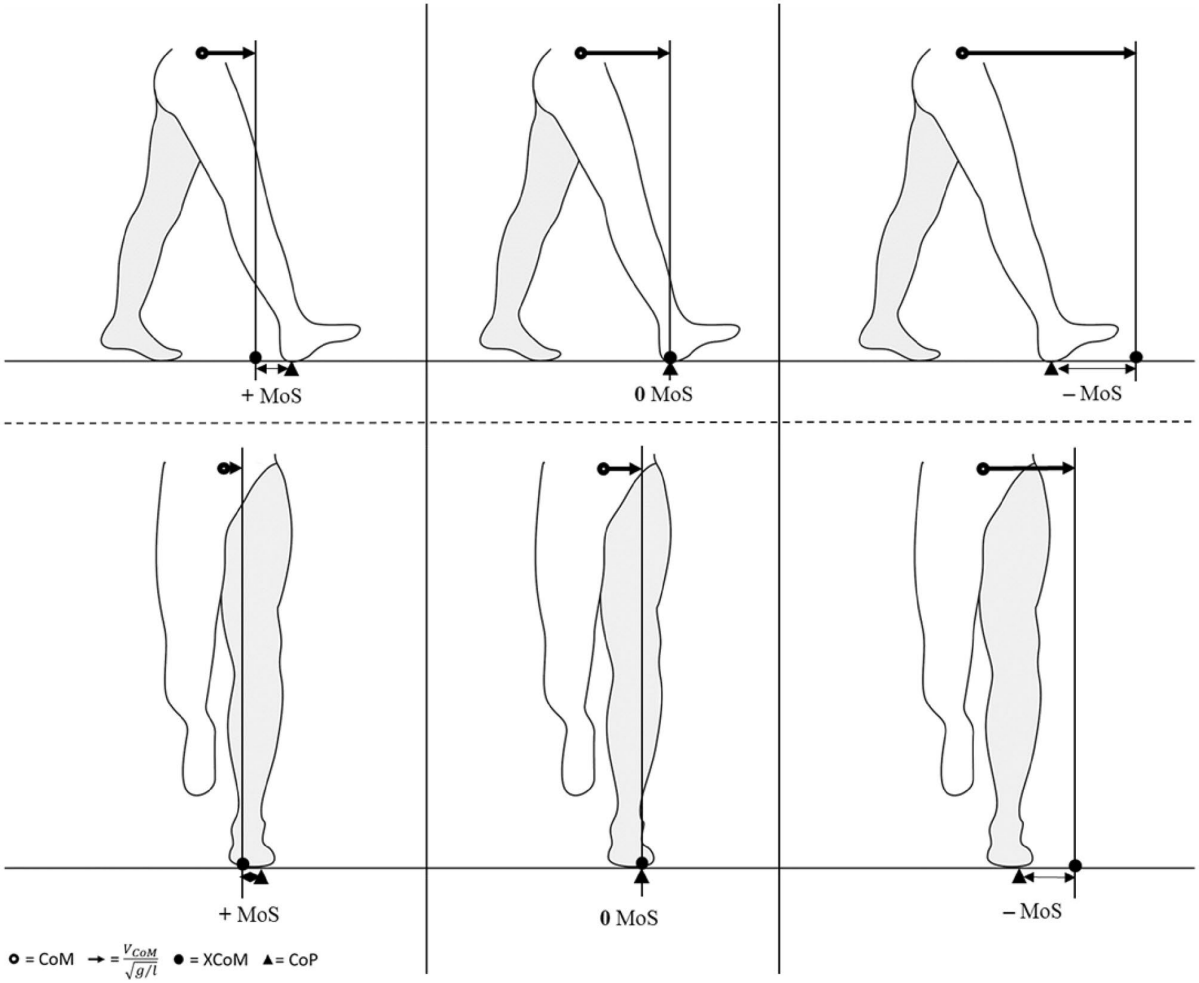
Schematic representation of the spatial margins of stability (MoS) in anterior–posterior (AP) direction at foot contact (top row), and medio-lateral (ML) direction during single support (bottom row). Left: The extrapolated centre of mass (XCoM) falls behind and medially of the centre of pressure (CoP), i.e., within the base of support, leading to a positive margin of stability (MoS) and resulting in the CoM reversing its motion if no further action is taken; Middle: the XCoM is projected right above the CoP, resulting to a MoS of zero, leading to the CoM coming to a halt; Right: The XCoM is projected in front and laterally of the CoP, thus resulting in a negative MoS, allowing the CoM to continue its motion. This figure has been adapted from Hak, et al.^16^ 10.1371/journal.pone.0082842.g001 in compliance with the CC BY 4.0 license.

One way to quantify stability of the body configuration during movement in this context, is to relate the CoM position, accounting for its velocity (i.e., the extrapolated centre of mass; XCoM), to the BoS^13,14^. The margins of stability (MoS;^15^), defined as the difference between XCoM and BoS can be calculated in both anterior–posterior (AP) and medio-lateral (ML) directions and indicates for a given time point whether the CoM will continue, halt or reverse its motion if no further action is taken (e.g., taking a step) or external forces are applied (Fig. 1)^16,17^. Steady-state walking (i.e., consistent forward progression) can simply be defined as the consistent placement of the centre of pressure (CoP) behind and outward of the XCoM at the time of foot contact, as stated by Hof^18^. This leads to mechanical instability in the anterior direction (i.e., a negative AP MoS) allowing the inverted pendulum to swing forward. When the CoP is placed in front of the XCoM (positive AP MoS), for example when walking slowly, this will increase anterior stability but require an “active” forward push of the CoM, rather than a “passive” forward swing of the CoM due to the pendulum dynamics. For the ML MoS, placing the CoP to the outside of the XCoM results in lateral stability and medial instability, which is compensated by placing the next step to the other side of the XCoM^16,19^.

Another way to quantify the stability of walking is by investigating spatiotemporal gait parameters and their step-to-step repeatability or variability^20,21^. Due to the impaired vestibular information processing in BVP, and vestibular feedback being essential in maintaining dynamic stability through the fine-tuning of timing and magnitudes of foot displacements, the gait disorder in BVP is primarily characterized by an increased gait variability and a higher risk of falls^22–26^. Additionally, BVP-patients with a history of falls exhibited a lower preferred walking speed with an increased step width and prolonged double support phase^27^.

Both the spatiotemporal gait parameters and the MoS are very closely related to each other as the MoS uses the BoS, which is related to the step length and width, as well as the CoM velocity, which is very closely related to walking speed, within its calculations^15^. Consequently, numerous theoretical and experimental studies have established interactions between spatiotemporal gait parameters and MoS, although primarily in healthy subjects^16,18,28–31^. As sensory feedback, and especially information from the vestibular system, plays a fundamental role in gait, the CoM trajectory is negatively affected whenever the vestibular system is impaired^32^. As a result, an impaired stability control during walking can be expected, which has an important influence on the capability of preventing a potential fall.

Therefore, during the present exploratory study, we analysed the gait of BVP-patients and healthy controls to further investigate the influence of an impaired peripheral vestibular system on gait. We aimed to determine the effect of a loss of peripheral vestibular function by comparing the spatiotemporal gait parameters and their step-to-step variability between BVP-patients and healthy controls during overground walking at preferred walking speed. We hypothesize that, in accordance with previous studies^24,26,27,33,34^, BVP-patients will show an increased gait variability during walking. Additionally, we also examined the interaction between the spatiotemporal gait parameters and MoS in BVP as compared to the healthy controls. We hypothesize that patients with BVP will regulate the MoS through controlling the state of the CoM, as opposed to altering the spatiotemporal gait parameters.

## Results

### Subject characteristics

A total of 27 BVP-patients were recruited. However, seven BVP-patients were excluded from the analysis as three patients presented with a central etiology (i.e., Lyme disease, cerebellar malaria, and encephalitis), two patients were only capable of walking with an assistive device and in two patients signal was lost during data acquisition. As a result, data of 20 BVP-patients were included and compared to data of 20 HCs. Groups were matched on age, sex and body mass and height. The subject characteristics are presented in Table 1. No significant differences were found between groups for age (p = 0.976), body mass (p = 0.978), body height (p = 0.929), leg length (p = 0.929) and BMI (p = 0.881).

**Table 1:** Descriptive data of the study sample including mean ages and anthropomorphic characteristics.

Parameter	Bilateral vestibulopathy (n = 20)			Healthy controls (n = 20)			p values
	Mean	SD	Range	Mean	SD	Range	
Age (years)	58.32	10.45	33.37–74.05	58.42	10.41	33.71–74.31	0.976
Body mass (kg)	79.2	14.8	57.00–111.00	79.33	14.14	47.50–99.60	0.978
Body height (m)	1.71	0.10	1.53–1.87	1.71	0.11	1.52–1.89	0.929
Leg length (m)	0.91	0.05	0.81–0.99	0.91	0.06	0.80–1.00	0.929
BMI	26.84	3.39	20.61–32.61	27.00	3.56	20.43–34.26	0.881
Sex (females)	6			6			
Time since onset (years)	9.45	8.04	1–30				

N = number of subjects; kg = Kilograms; m = metre.

p-values are calculated using a 2-sample *t* test.

### Gait parameters

The gait parameter results are presented in Table 2. Significant differences were found between BVP-patients and healthy controls. BVP-patients showed a higher mean cadence (121 ± 9 vs 115 ± 8 steps/min; p = 0.020, *d* = 0.77) and lower step time (0.50 ± 0.04 vs 0.53 ± 0.03 s; p = 0.020, *d* = − 0.77) compared to HCs. No significant differences were found between BVP-patients and healthy controls in walking speed (1.26 ± 0.19 vs 1.22 ± 0.18 m/s, p = 0.505), step length (0.63 ± 0.10 vs 0.64 ± 0.08 m; p = 0.882) and step width (0.18 ± 0.04 vs 0.17 ± 0.03 m; p = 0.370). Also, single- and double support phases did not differ between BVP-patients and HCs: 40 ± 2 vs 40 ± 2%GC (p = 0.989) and 21 ± 4 vs 22 ± 3%GC (p = 0.999) respectively. As for variability parameters, no significant differences were found between groups. Although the difference in step width CoV did not reach significance (19 ± 11% for BVP versus 13 ± 5% for HCs; p = 0.072), a moderate effect size of *d* = 0.60 was found.

**Table 2:** Means and standard deviations of the spatiotemporal gait parameters, their variability (coefficient of variation), and spatial margins of stability in the bilateral vestibulopathy patients and healthy controls.

	Bilateral vestibulopathy (n = 20)			Healthy controls (n = 20)			p values	Cohen’s d
	Mean	SD	95% CI	Mean	SD	95% CI		
**Gait parameters**								
Walking speed (m/s)	1.26	0.19	[1.17;1.35]	1.22	0.18	[1.14;1.30]	0.505	0.21
Cadence (steps/min)	121	9	[117;126]	115	8	[111;118]	0.020*	0.77
Step time (s)	0.50	0.04	[0.48;0.52]	0.53	0.03	[0.51;0.54]	0.020*	− 0.77
Step length (m)	0.63	0.10	[0.58;0.67]	0.64	0.08	[0.60;0.68]	0.882^†^	0.05
Step width (m)	0.18	0.04	[0.16;0.20]	0.17	0.03	[0.16;0.18]	0.370	0.29
Single support phase (%GC)	39.46	2.16	[38.45;40.47]	39.47	1.47	[38.78;40.15]	0.989	0.00
Double support phase (%GC)	21.05	4.14	[19.11;22.99]	21.05	2.94	[19.68;22.42]	0.999	0.00
Walking speed CoV (%)	4.29	3.23	[2.78;5.80]	4.81	1.78	[3.97;5.64]	0.126^†^	0.50
Cadence CoV (%)	3.87	2.99	[2.47;5.27]	3.92	1.58	[3.18;4.66]	0.199^†^	0.42
Step time CoV (%)	3.77	2.74	[2.49;5.05]	3.88	1.51	[3.17;4.59]	0.229^†^	0.39
Step length CoV (%)	4.95	4.95	[2.64;7.27]	3.72	1.29	[3.11;4.32]	0.675^†^	0.14
Step width CoV (%)	18.88	11.11	[13.68;24.08]	12.75	5.28	[10.29;15.22]	0.072^†^	0.60
Single support phase CoV (%)	3.74	2.55	[2.55;4.93]	3.83	1.55	[3.10;4.55]	0.250^†^	0.37
Double support phase CoV (%)	7.15	3.84	[5.35;8.94]	9.81	5.85	[7.07;12.55]	0.148^†^	0.48
**Spatial margins of stability**^**$**^								
AP MoS (m)	− 0.14	0.04	[− 0.12; − 0.17]	− 0.20	0.04	[− 0.18; − 0.22]		
ML MoS (m)	0.05	0.02	[0.05;0.06]	0.05	0.01	[0.04;0.05]		

m/s = metres per second; steps/min s steps per minute; s = seconds; m = metre; %GC = percentage of gait cycle; CoV = Coefficient of Variation; AP MoS = Anterior–posterior Margin of Stability; ML MoS = medio-lateral Margin of Stability.

Underlined values represent non-normally distributed parameters.

^†^Compared using the Mann–Whitney’s *U* test.

*Significant at the 0.05.

^$^MoS are not compared.

### Partial least squares analysis

#### Relationship between gait variables, age, and BMI versus AP MoS

The PLS model for BVP (Table 3), consisting of 1 latent factor, explained 65% of variance of the AP MoS. The variables with a VIP > 1.0 were walking speed (VIP = 1.033; β = -0.290) and double support phase (VIP = 1.009; β = 0.280). Single support phase showed the lowest VIP (0.965) with a regression coefficient of − 0.270. Based on the VIP and regression coefficients, walking speed together with double support phase can be noted as prime predictors for the AP MoS. An Actual by Predicted plot can be found as Supplementary Fig. 1a.

**Table 3:** Results of the partial least squares model for the AP MoS in BVP-patients.

	PRESS	# LF’s	X Variation explained	Y Variation explained	Q^2^	R^2^*Y*	β_O_	β_S_	VIP
Model	0.693	1	92.45%	65.12%	0.52	0.65	− 144.118	0.000	
Walking speed (m/s)							− 12.620	− 0.290	1.033
Single support phase (%GC)							− 11.777	− 0.270	0.965
Double support phase (%GC)							12.223	0.280	1.009

AP MoS: Anterior–posterior margin of stability; PRESS: Predictive Residual Sum of Squares; # LF’s: number of Latent Factors; Q^2^: goodness of prediction; R^2^*Y*: goodness of fit; β_O_: regression coefficient for original data; β_S_: regression coefficient for centred and scaled data; VIP: Variable Importance in Projection; m/s: metres per second; %GC: percentage of gait cycle.

For the HCs, the PLS model (Table 4) consisted of 3 latent factors, explaining 76% of the variance in AP MoS. The only variable with a VIP > 1.0 was walking speed (VIP = 1.197; β = − 1.540). Cadence, step time and step length were less important in the prediction of the AP MoS (VIP < 1.0). An Actual by Predicted plot can be found as Supplementary Fig. 2a.

**Table 4:** Results of the partial least squares model for the AP MoS in HCs.

	PRESS	# LF’s	X Variation explained	Y Variation explained	Q^2^	R^2^*Y*	β_O_	β_S_	VIP
Model	0.596	3	99.98%	76.20%	0.65	0.76	− 204.153	0.000	
Walking speed (m/s)							− 65.847	− 1.540	1.197
Cadence (steps/min)							− 55.755	− 1.304	0.942
Step time (s)							− 60.528	− 1.416	0.923
Step length (m)							32.459	0.759	0.910

AP MoS: Anterior–posterior margin of stability; PRESS: Predictive Residual Sum of Squares; # LF’s: number of Latent Factors; Q^2^: goodness of prediction; R^2^*Y*: goodness of fit; β_O_: regression coefficient for original data; β_S_: regression coefficient for centred and scaled data; VIP: Variable Importance in Projection; m/s: metres per second; %GC: percentage of gait cycle.

#### Relationship between gait variables, age, and BMI versus ML MoS

The PLS model for BVP (Table 5), consisting of 4 latent factors, explained 87% of variance in the ML MoS. Only step width achieved a VIP > 1.0 with a regression coefficient (β) of 0.970. Walking speed, step length, single support phase, and double support phase showed VIP scores ranging between [0.819;0.885] with regression coefficients between [− 0.698;0.769]. An Actual by Predicted plot can be found as Supplementary Fig. 1b.

**Table 5:** Results of the partial least squares model for the ML MoS in BVP-patients.

	PRESS	# LF’s	X Variation explained	Y Variation explained	Q^2^	R^2^*Y*	β_O_	β_S_	VIP
Model	0.486	4	99.89%	86.87%	0.76	0.87	51.809	0.000	
Walking speed (m/s)							11.587	0.769	0.885
Step length (m)							− 10.507	− 0.698	0.855
Step width (m)							14.605	0.970	1.464
Single support phase (%GC)							− 3.631	− 0.241	0.819
Double support phase (%GC)							− 2.128	− 0.141	0.819

ML MoS: Medio-lateral margin of stability; PRESS: Predictive Residual Sum of Squares; # LF’s: number of Latent Factors; Q^2^: goodness of prediction; R^2^*Y*: goodness of fit; β_O_: regression coefficient for original data; β_S_: regression coefficient for centred and scaled data; VIP: Variable Importance in Projection; m/s: metres per second; %GC: percentage of gait cycle.

In HCs, the model consisted of 1 latent factor (Table 6), explaining 65% of variance of the ML MoS. The only variable included was step width with a VIP of 1.000 and a regression coefficient of 0.808. An Actual by Predicted plot can be found as Supplementary Fig. 2b.

**Table 6:** Results of the partial least squares model for the ML MoS in HCs.

	PRESS	# LF’s	X Variation explained	Y Variation explained	Q^2^	R^2^*Y*	β_O_	β_S_	VIP
Model	0.644	1	100%	65.34%	0.59	0.65	46.706	0.000	
Step width (m)							8.296	0.808	1.000

ML MoS: Medio-lateral margin of stability; PRESS: Predictive Residual Sum of Squares; # LF’s: number of Latent Factors; Q^2^: goodness of prediction; R^2^*Y*: goodness of fit; β_O_: regression coefficient for original data; β_S_: regression coefficient for centred and scaled data; VIP: Variable Importance in Projection; m: meter.

## Discussion

The purpose of this study was to examine the influence of an impaired vestibular system, due to a bilateral loss of peripheral vestibular function, on spatiotemporal gait parameters during overground walking at preferred walking speed. The results of the study indicate that BVP-patients primarily walk with an increased cadence, while other mean spatiotemporal parameters did not differ with the healthy controls. Concerning the variability in spatiotemporal parameters, no significant differences were found between both populations, although a moderate effect size was noted for step width variability (bilateral vestibulopathy: 19 ± 11%; healthy controls: 13 ± 5%). Thus, the hypothesis of BVP-patients exhibiting an increased gait variability can neither be confirmed, nor completely rejected, based on the present results. In addition, results of the PLS indicated that, close to walking speed, the double support phase was the prime predictor for the AP MoS in BVP-patients, while for HCs this was walking speed alone. For the ML MoS, in both populations, step width was the prime predictor. Therefore, mechanical stability in the AP direction was managed differently in BVP-patients compared to HCs but did not differ for the ML direction, partially confirming our hypothesis.

### Interpretation

The current study provides a first look into how gait performance and stability is affected by the bilateral loss of vestibular function during steady state overground walking. Our findings of BVP-patients exhibiting an increased cadence with a self-selected walking speed comparable to healthy subjects may be explained through previous findings indicating a selective suppression of the vestibular influence on lower limb muscles with increased cadence and walking speed^22,35^. Therefore, we could reason that BVP-patients choose not to walk at a slower speed, but instead retain the same walking speed as healthy subjects. Although BVP-patients present with a higher cadence, no difference in step length was found as would be expected but could be attributed to the limited sample size. However, the increase in cadence can point towards the suppression of inaccurate, or even completely missing, vestibular information. This could indicate a shift from a sensory feedback-driven control of balance to a more feedforward control of balance, as has been suggested before^22,35–37^. In addition, it has been shown that during slow walking speeds, the variability of most parameters is highest in BVP-patients^24,26,27,34,38^. The reason for this could be an increased need for sensory feedback at slower walking speeds in general, as passive dynamics play a smaller role in stabilising the body during this condition. The evidence of vestibular suppression at faster walking speeds as described above further supports this point.

Concerning the relationship between the AP MoS and spatiotemporal parameters, in both populations walking speed was extracted as a variable predicting the AP MoS. As the AP MoS is directly dependent on the walking speed^39,40^, it seems sensible that walking speed is extracted. However, as indicated by the PLS analysis, single support and double support phases seem to be important predictors for the AP MoS in BVP-patients as well, compared to cadence, step time, or step length found in the HCs. The regression coefficient of the double support phase implies an increased (i.e., less negative) AP MoS with a longer double support phase, and a decreased (i.e., more negative) AP MoS with a longer single support phase in BV-patients. This could imply a strategy to primarily control the CoM state, instead of altering the walking speed or any of the underlying step parameters^16,28,41–43^.

Regarding the control of the CoM during human walking, the CoM state (e.g., position, velocity) is most effectively influenced during the stance phases, i.e. the single and double support phase. During single support phases the CoM is supported with very little muscle force or work^44,45^. This energy-conserving motion is suddenly interrupted by the collision of the swing leg with the ground, changing the velocity of the CoM^44^. Therefore, during the double support phase the CoM velocity must be redirected, from one inverted pendulum to the next, in order to prepare for the next step and to maintain a steady progression^44^. Through alterations in the forces applied on the ground, the direction of the CoM acceleration can be changed in order to control the progression as well as the dynamic equilibrium^23^. Considering the integration of both proprioceptive information and vestibular input during the double support phase^23^, it seems appropriate that BVP-patients would prefer for the double support phase to control the state of the CoM. This integration provides the opportunity to specify whether the movement of the body relative to the BoS will result in the desired end position^23^. However, due to the lack of reliable vestibular information, BVP-patients will need to rely primarily on the incoming proprioceptive information, and additionally also the visual information.

Moreover, BVP-patients may adapt the way the CoM is controlled to promote gaze stability and consequently increase the reliability of incoming visual information. As an impaired gaze stability during walking (i.e., oscillopsia) is reported in 50% to 70% in BVP-patients^3^, an appropriate gaze stabilization may also improve gait stability^46,47^. In healthy adults, a head-in-space stabilization strategy during gait is used to facilitate the integration of visual and vestibular information for motor control of the ongoing locomotor task^48,49^, while in BVP-patients, a strategy to stabilize the head on the trunk seems to be used as reported by Pozzo, et al.^49^. This head-on-trunk stabilization may be used to improve gaze fixation on a visual target which serves as an anchor point to base the head position in space^47^. In addition to the improved gaze fixation, a head-on-trunk stabilization also results in a single body segment consisting of the head and trunk^50^. While this is beneficial for the central nervous system as it simplifies the motor control of the body segments, a single large segment (i.e., head and trunk combined) is more stable and more difficult to disturb, however is more difficult to control when successfully perturbed, due to the increased mass and consequential inertia. Hence, current results may point towards a control strategy where BVP-patients control the CoM instead of altering the spatiotemporal parameters to obtain a stable way of walking.

However, the reasoning described above may not be applicable for the medio-lateral balance control of gait as it has been suggested that the medio-lateral foot placement still requires vestibular information, even at increased walking speeds^33,34^. During the swing phase, the foot placement is determined and coordinated, however due to only one foot being in contact with the ground, the available proprioceptive information is reduced. As a result, the additional lack of vestibular input may therefore decrease the accuracy of foot placement, translating in an increased step width variability, and may thus indicate the importance of vestibular input during the swing phase^34^. As compared to HCs, where step width was the only variable extracted as predictor for the ML MoS, the model for ML MoS in BVP-patients also included walking speed, step length, and the single and double support phases. Single support phase was also extracted as predictor for the ML MoS. When considering the inverted pendulum, a shorter time spent on one leg, in addition to a faster transition to a more stable double support phase, also results in a reduced excursion of the CoM. This reduced excursion effectively increases the minimum distance between the XCoM and CoP resulting in an increase in ML MoS^51^. Although the single support phase can also play a part in determining the ML MoS, step width was still extracted as the primary predictor in both BVP-patients as in HCs. Altogether, the inclusion of these additional variables in the model of BVP-patients did result in an increased goodness of fit and goodness of prediction as compared to the model of HCs.

### Limitations and future research

Some limitations are to be kept in mind. All HCs participated on a voluntary basis which may induce a possible selection bias. Additionally, HCs were only excluded based on self-reported visual, neurological or orthopaedic disorders and were thus not screened on any cognitive, mental or cardiovascular disorders which could coincidentally affect gait^52,53^. BVP-patients were currently only included when they were able to walk without assistance, which might place the current investigated patient population at a higher functional level as the populations investigated in previous studies^24,26,27,34,38^. Thus the current results might not be generalisable to patients that are less mobile. For example, da Silva, et al.^54^ also found, albeit being in a population of individuals with visual impairment, that those individuals who participated in disability sports (i.e., were at a higher functional level) had a similar self-selected walking speed as active sighted individuals. These results also differed from previous studies investigating individuals with visual impairments with a lower physical activity level.

With regards to the reduced marker model used for the BVP-patients, this model has only been validated for treadmill walking^39,40,55^. Additionally, instead of using the toe and the anterior border of the base of support, the ankle marker was used which results in a systematic overestimation of the AP MoS in both BVP-patients and HCs. Therefore, we caution comparing the numeric values of the AP MoS reported in this study to those using the toe and anterior boundary. Also, as the reduced marker model used with BVP-patients includes the major trochanter as opposed to the Superior Anterior Iliac Spine in the Plug-In-Gait marker model used in the HCs, this results in a systematic difference in MoS. This difference in marker placement resulted in an altered position of the CoM between the reduced model and the Plug-in-Gait model, and consequently resulting in a different magnitude of the AP MoS (Supplementary Fig. 3). The reduced model also does not fully capture the effects of pelvic motions as opposed to the Plug-in-Gait model which may limit the estimation accuracy of the body’s centre of mass trajectory. For an interesting paper discussing the estimation accuracy of the body’s 3D centre of mass trajectory during walking using different marker models we refer to Pavei, et al.^56^.

Furthermore, the number of steps used to calculate the variability parameters were rather limited. The total range of steps available in BVP-patients ranged from 4 to 14 steps in total, while in HCs numbers ranged from 6 to 22 steps as the walkway had a length of 12 m, with only the middle 6 m being used for analysis. Although the total number of steps used to calculate the variability seems low, step-to-step variability can be reliably assessed using less than 15 steps^57,58^.

Additionally, it would be of great interest to look further into the ground reaction forces generated during the single and double support phases and compare these between BVP-patients and HCs. This would enable to test the hypothesis where BVP-patients rely more on the control of the CoM through altering the forces generated during the single and double support phases, instead of altering the underlying step parameters. A graphical representation of the ground reaction forces of both the BVP-patients and the healthy controls can be found as supplementary information (Supplementary Figs. 4-6). These results were not included in the present manuscript as they fall outside the scope of this study. However, this information may be of importance for future research concerning this matter.

Lastly, looking into the possibility whether gait parameters or the margins of stability can distinguish between patients with different functional impairments, or fallers versus non-fallers. This could aid the development of diagnostic test protocols on a functional level, both in normal ageing and in different pathologies.

## Conclusion

In conclusion, BVP-patients seem to implement a balance control strategy where they prefer to directly control the state of the CoM itself instead of the underlying step parameters. This is suggested by the lack of differences in spatiotemporal gait parameters with healthy controls, with only cadence showing a significant difference. Additional indications may be how the mechanical stability in anterior–posterior and medio-lateral direction is controlled in BVP-patients as compared to healthy controls, where BVP-patients may rely more on the single and double support phases to control the state of the CoM.

## Methods

### Study design

Data on 3D gait analysis in a convenience sample of 20 patients with BVP and 20 healthy controls (HCs), matched by age, sex, body height and weight, were included in this cross-sectional study. Spatiotemporal parameters and gait stability parameters (MoS) were investigated in both groups. The study protocol has been approved by the local Ethics Committee of the University of Antwerp / Antwerp University Hospital (B300201629697), with data collection of BVP-patients taking place between December 2017 and October 2018. Data of healthy controls were retrospectively selected from a database containing 114 subjects, collected between April 2015 and January 2016. Subjects gave written informed consent at the time of the study inclusion and were aware that data could be used retrospectively for further research which was approved by the local Ethics Committee (B300201316328). This study was conducted in accordance with the Declaration of Helsinki.

### Setting

An instrumented gait analysis was performed using a three-dimensional motion capture system with eight cameras (Vicon T10, 100 Hz., Vicon Motion Systems Ltd., Oxford, UK, 100 fps, resolution 1 Megapixel (1120 × 896), 3 AMTI type OR 6–7 force plates (1000 fps, 46 × 50 × 8 cm) and 1 AccuGait (1000 fps) force plate. Reflective markers were placed on anatomical landmarks on the subject’s body based on the reduced marker model as described by Süptitz, et al.^55^, corresponding to the left and right first metatarsal (toe), major trochanter, the sacrum, and C7, with addition of the lateral malleoli (ankle), for the BVP-patients. The ankle markers were added in the current study to determine the foot touchdown more precisely. For the healthy subjects, reflective markers were placed according to the full-body Plug-In-Gait model^59^, consisting of 4 markers on the head, 4 on the torso, 10 on the upper limb, 5 on the pelvis, and 12 on the lower limb, resulting in a total of 35 markers on different anatomical landmarks.

### Data measurement and calculations

Both BVP-patients and HCs walked barefoot over a 12-m-long walkway at a preferred walking speed, with the middle 6 m used for data collection. Data of BVP-patients was collected during the performance of the first item of the functional gait assessment (i.e. gait on a level surface). The total number of steps ranged from 4 to 14 steps. Data collection of the healthy controls has been described extensively in Herssens, et al.^31^. The total number of steps for the included HCs ranged from 6 to 22 steps. Marker trajectories were tracked and labelled using the Vicon Nexus 2.x.x software for both BVP-patients and HCs. Based on a combination of force plate data (20 N threshold) and ankle and toe marker trajectories, events of foot strike and foot off were determined using the “Dynamic Plug-in Gait Pipeline”^60^. Gait cycles were calculated based on left and right ankle marker trajectories.

The .c3d files were exported to Matlab (R2019a for Windows) and through a custom-made script, the spatiotemporal parameters were calculated from the left and right ankle marker trajectories, the margins of stability were calculated as described below.

### Participants

BVP-patients were included according to the diagnostic criteria suggested by the Bárány Society^9^. Participants were excluded if they presented with a central etiology, they could not walk without assistance (e.g. the use of an assistive device or another person) or had any other known neurological or orthopaedic comorbidities that could influence motor performances. Information on vestibular function testing of all included BVP-patients can be found in Supplementary Table 1.

Healthy controls were matched by age, sex and body height and did not report any visual impairments, antalgic gait pattern, abnormal mobility in the lower limbs or any known neurological or orthopaedic disorder that could influence motor performance and balance^31^.

### Variables of interest

#### Subject characteristics

For each subject, information concerning age (years), body mass (kg), body height (cm) and BMI ($body \,mass \left( {kg} \right)/body\, height (m)$) were obtained.

For BVP-patients the time since onset of symptoms (years) on the day of assessment was calculated. Information on diagnosis and vestibulo-ocular reflex function testing can be found in Supplementary Table 1.

#### Gait parameters

Walking speed (ms^-1^; calculated as stride length divided by stride time), cadence (steps/min), step time (s), -length (m) and -width (m), and the duration of double and single support phase (%) were collected. Means and Coefficients of Variation (CoV; variability) were calculated over the total amount of steps recorded. The CoV was calculated as follows: $CoV = \left( {\frac{Standard\, deviation}{{Mean}}} \right) \times 100\%$. By calculating the Coefficient of Variation, the variance around the mean is described. Although the total number of steps used to calculate the variability seems low, step-to-step variability can be reliably assessed using less than 15 steps^57,58^. Gait parameters were considered as absolute values.

#### Extrapolated centre of mass

The extrapolated centre of mass (XCoM) is defined as the vector sum of the centre of mass position and a proportion of its velocity as described by Hof, et al.^15^. The XCoM used in this study was adapted to the reduced kinematic model^55^ used for the BVP-patients, as described above. The current method differs from that of Hof, et al.^15^, as in the current study the position of the centre of mass (CoM) was estimated by using the average of the markers placed on the major trochanter for the reduced model (BVP-patients) or Anterior Superior Iliac Spine for the Plug-In-Gait model (HCs) (Supplementary Fig. 1).

$$XCoM = \frac{{P_{m1} + P_{m2} }}{2} + \frac{{0.5\left( {\frac{{v_{m1} + v_{m2} }}{2} + v_{C7} } \right)}}{{\sqrt {{\raise0.7ex\hbox{$g$} \!\mathord{\left/ {\vphantom {g l}}\right.\kern-\nulldelimiterspace} \!\lower0.7ex\hbox{$l$}}} }}$$

With $P_{m1}$, $P_{m2}$ corresponding to the positions of the left and right major trochanter marker positions in the reduced model and the left and right Anterior Superior Iliac Spine marker positions in the Plug-In-Gait model, representing the vertical projection of the CoM. $v_{m1}$, $v_{m2}$, and $v_{C7}$ are the velocities of the major trochanter/Anterior Superior Iliac Spine and C7 markers respectively, representing the velocity of the CoM. Lastly, $g$ represents the acceleration of gravity (9.81 ms^-2^) and $l$ is defined as the leg length defined as a fraction of body height: $0.530*Body height \left( {mm} \right)$^61^.

### Spatial margins of stability

The MoS were calculated based on the equations defined by Hof, et al.^15^. The medio-lateral MoS was defined in this study as the minimum distance between the boundary of the BoS, i.e. the ankle marker ($P_{Ankle}$), and the XCoM along the medio-lateral axis during the single support phases. The medio-lateral axis was defined as the axis in the transverse plane, perpendicular to the walking direction derived from the CoM coordinates.

$$ML MoS = P_{Ankle} - XCoM$$

The anterior–posterior MoS was defined in this study as the distance between the boundary of the BoS of the leading foot, i.e. the ankle marker ($P_{Ankle}$), and the XCoM along the anterior–posterior axis at foot touchdown. With the anterior–posterior axis being defined as the axis in the transversal plane, parallel to the walking direction derived from the CoM coordinates.

$$AP MoS = P_{Ankle} - XCoM$$

### Statistical analysis

Statistical analysis was performed using JMP Pro statistical software (version 14 for Windows, SAS Institute). To describe the population, means, standard deviations of subject characteristics and gait parameters were calculated. Normality of both samples was checked using the Shapiro–Wilk test for normality and QQ-plots. In case both samples passed the Shapiro–Wilk test the two-sample *t* test (two-sided) was conducted, otherwise the Mann–Whitney *U* test was used to compare both samples. Differences were significant at the 0.05 level, and effect size was estimated using Cohen’s *d* test statistics, using the methods described in Lenhard and Lenhard^62^. An effect size *d* >|0.5| was considered relevant as this may represent “an effect likely to be visible to the naked eye of the observer”^63,64^. Spatial margins of stability were not compared between both samples as the difference in marker model used for BVP-patients and HCs results in a systematic difference, especially in the AP MoS (Supplementary Fig. 1).

Additionally, to explore the relationship between the mean gait parameters, BMI and age versus the margins of stability^31^, a Nonlinear Iterative Partial Least Squares (NIPALS) analysis with leave-one-out cross validation was performed. A PLS analysis can appropriately be applied to different shapes of data, for example when there are a greater number of observations relative to the variables (i.e., tall data); when the number of predictor and/or response variables are greater relative to the observations (i.e., wide data); or, when the number of observations and variables are equal (i.e., square data)^65–67^. In addition, a PLS analysis can also be used when the predictor variables are highly collinear^65,68^. In contrast to usual regression analysis, PLS controls for dependencies among gait outcomes and therefore enables to consider the data in an overarching way^68^. Some other advantages are that, while predictor variables need to be continuous, the response variables can be continuous or categorical. Additionally, PLS requires only few assumptions: the data should be relatively normally distributed and should be screened for possible influential outliers prior to running the analysis. To mitigate a non-normal distribution of the data, or in case of extreme outliers, one can perform a logarithmic transformation prior to analysis^65^. As step length was found to have a non-normal distribution in the BVP-patients, a logarithmic transformation was performed prior to running the PLS analysis.

In the current study, the number of observations (20 subject in each group) was greater relative to the variables included in the analysis (n = 9), thus resulting in “tall” data-shape. Additionally, important multi-collinearity was present among the independent variables. For example, changes in walking speed result in changes of step length and step time and vice versa. Dealing with this multi-collinearity is crucial, especially with respect to gait outcomes.

Using this PLS analysis, for each group, the internal covariance structure among the mean gait parameters, age, and BMI (X-factors) best modelling the AP and ML MoS (Y-response) was identified by removing common variance and by finding underlying latent factors (LF’s). Individual prediction models were built for the AP and ML MoS in both BVP-patients and HCs, respectively. The optimal number of LF’s is determined by adding LF’s until the Predicted Residual Sum of Squares (PRESS) is smallest. The percent of variance explained for X (factors) and Y (responses) by the LF’s indicated the modelling power of those outcomes. The Variable Importance in Projection (VIP) quantifies the importance of each variable in the final model. Variables with a VIP value > 1 are considered very important, whereas variables with a VIP < 0.8 have less influence on the model^68^. The regression coefficients (β) on the other hand represent the influence each variable (i.e., mean gait parameters, age, or BMI) has in the prediction of the response (i.e., AP or ML MoS). A large absolute coefficient, together with a VIP value > 1.0 indicates that a variable is a prime candidate in the model^68^. The variables with the lowest VIP are dropped in the first model and the PLS model is run again, continuing until all included variables have a VIP of 0.8 or greater. The percent of variation explained for X (factors) and Y (responses) were investigated, as were the Q^2^ and Cumulative R^2^*Y* values to determine the predictive quality of the model. The Q^2^-value shows the model’s predictive validity, thus shows how well the collected data can be reconstructed with the help of the model. When the Q^2^-value is greater than 0.5, the model is regarded as a predictive model^69^. As for the R^2^*Y*-values, these reflect the level of the latent construct’s explained variance, i.e., the “goodness of fit”^69^. Although no generalizable statement can be made about acceptable threshold values^70^, the larger the value, ranging between 0 and 1, the larger the percentage of variance explained^69^.

## Ethical approval

The study protocol has been approved by the local Ethics Committee of the University of Antwerp / Antwerp University Hospital (B300201629697). Subjects gave written informed consent at the time of the study inclusion and were aware that data could be used retrospectively for further research which was approved by the local Ethics Committee (B300201316328).

## Supplementary Information

Supplementary Information

## Data Availability

The dataset including data on subject characteristics, spatiotemporal parameters and margins of stability are publicly accessible in the figshare data repository at 10.6084/m9.figshare.12980468.v1
