# Supplementary material for: An exploratory investigation on spatiotemporal parameters, margins of stability, and their interaction in bilateral vestibulopathy
Source: Sci Rep. 2021 Mar 19;11:6427. doi: 10.1038/s41598-021-85870-7 (PMC7979710; doi:10.1038/s41598-021-85870-7)
Supplement: Supplementary file 1 — Supplementary Information [file 41598_2021_85870_MOESM1_ESM.docx]

# An exploratory investigation on spatiotemporal parameters, margins of stability, and their interaction in bilateral vestibulopathy.

Nolan Herssens^1-3^*, Wim Saeys^2,4^, Luc Vereeck^2,3^, Kenneth Meijer^5^, Raymond van de Berg^6,7^, Vincent Van Rompaey^8,9^, Christopher McCrum^5§^ & Ann Hallemans^2,3§^.

Corresponding author: Nolan Herssens, Campus UZ Gent, Corneel Heymanslaan 10, Building B3, 9000, Ghent, Belgium. [nolan.herssens@ugent.be](mailto:nolan.herssens@ugent.be), +32 4 74 22 29 62.

^1^Department of Rehabilitation Sciences, Ghent University, Ghent, Belgium

^2^ Department of Rehabilitation Sciences and Physiotherapy/Movant, Faculty of Medicine and Health Sciences, University of Antwerp, Belgium

^3^ Multidisciplinary Motor Centre Antwerp (M²OCEAN), University of Antwerp, Belgium.

^4^ RevArte Rehabilitation Hospital, Edegem, Antwerp.

^5^ Department of Nutrition and Movement Sciences, NUTRIM School of Nutrition and Translational Research in Metabolism, Maastricht University Medical Centre+, Maastricht, The Netherlands.

^6^ Division of Balance Disorders, Department of Otorhinolaryngology and Head and Neck Surgery, Faculty of Health Medicine and Life Sciences, School for Mental Health and Neuroscience, Maastricht University Medical Centre+, Maastricht, The Netherlands

^7^ Faculty of Physics, Tomsk State University, Tomsk, Russia.

^8^ Department of Otorhinolaryngology and Head & Neck Surgery, Antwerp University Hospital, Edegem, Belgium

^9^ Faculty of Medicine and Health Sciences, University of Antwerp, Belgium.

§ Christopher McCrum and Ann Hallemans should be considered joint senior author as they contributed equally to this manuscript.


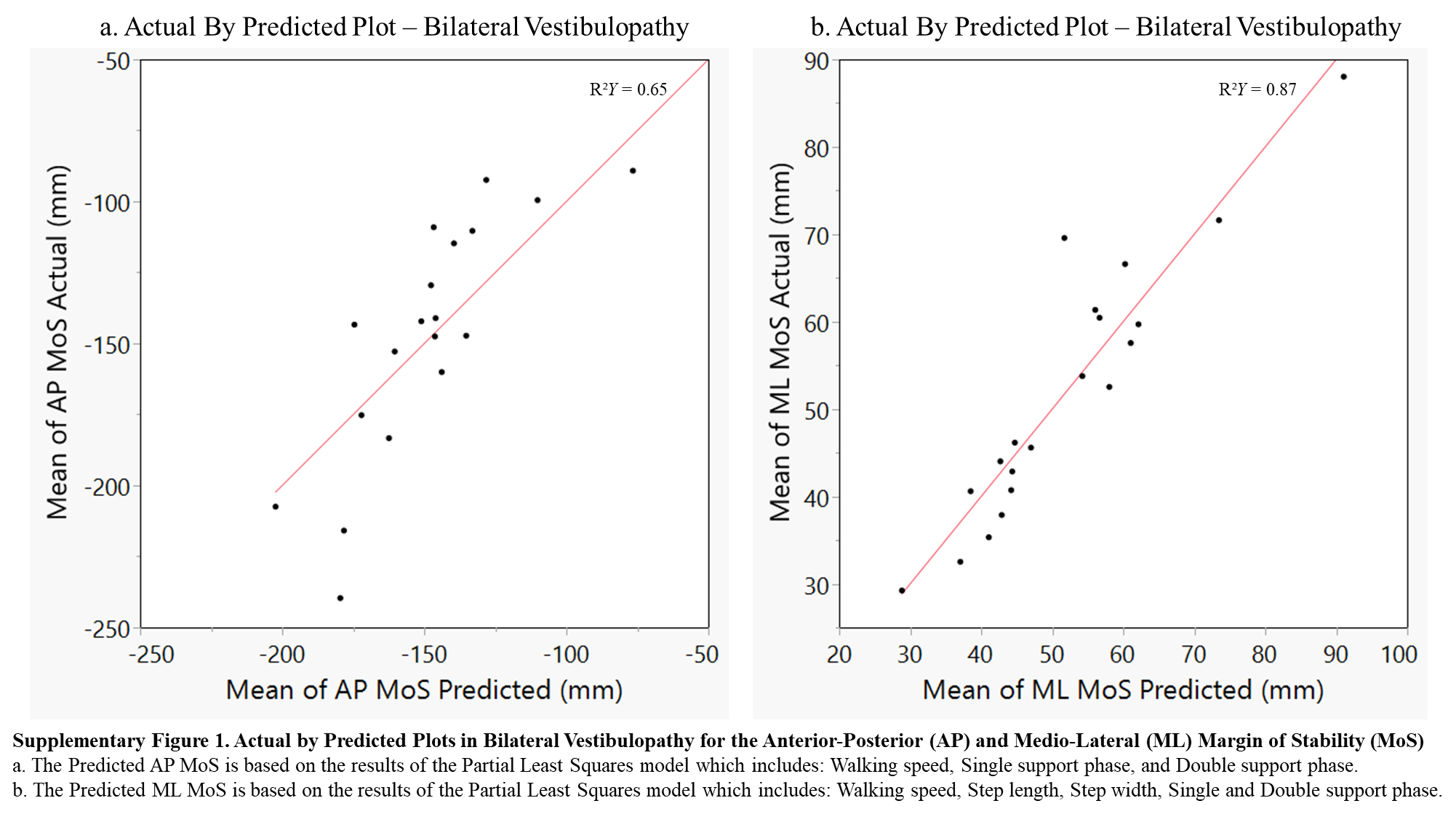


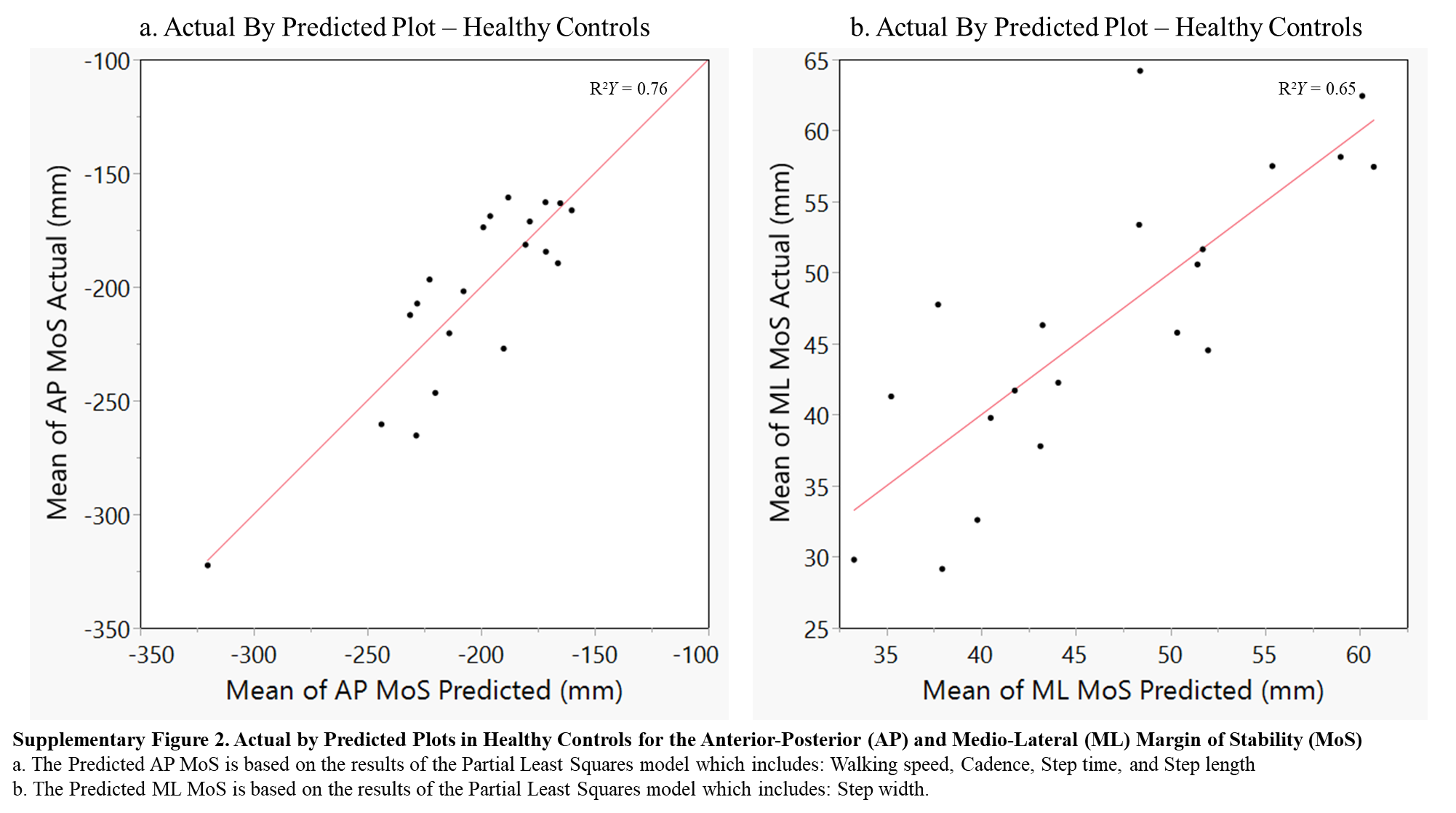


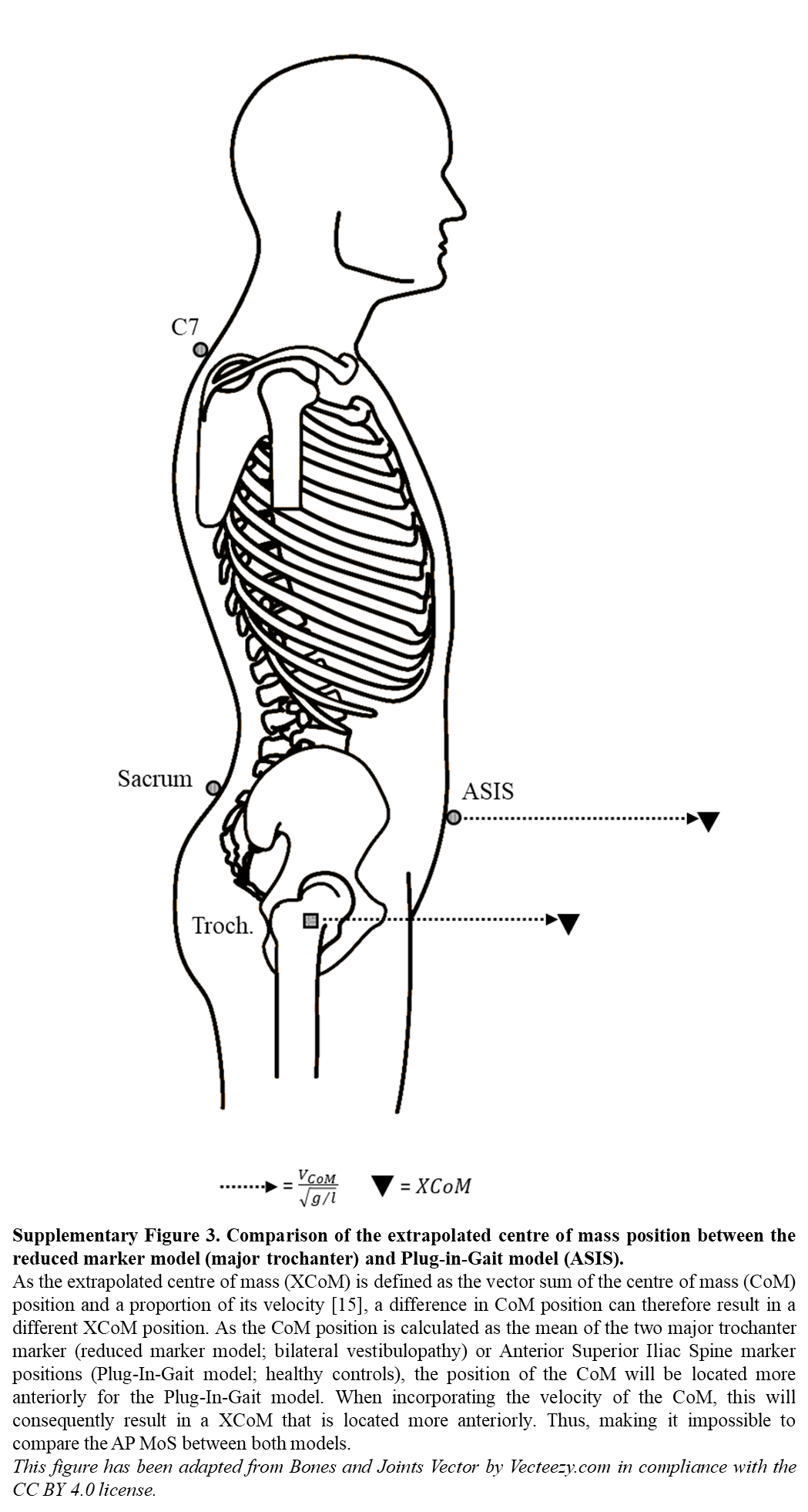


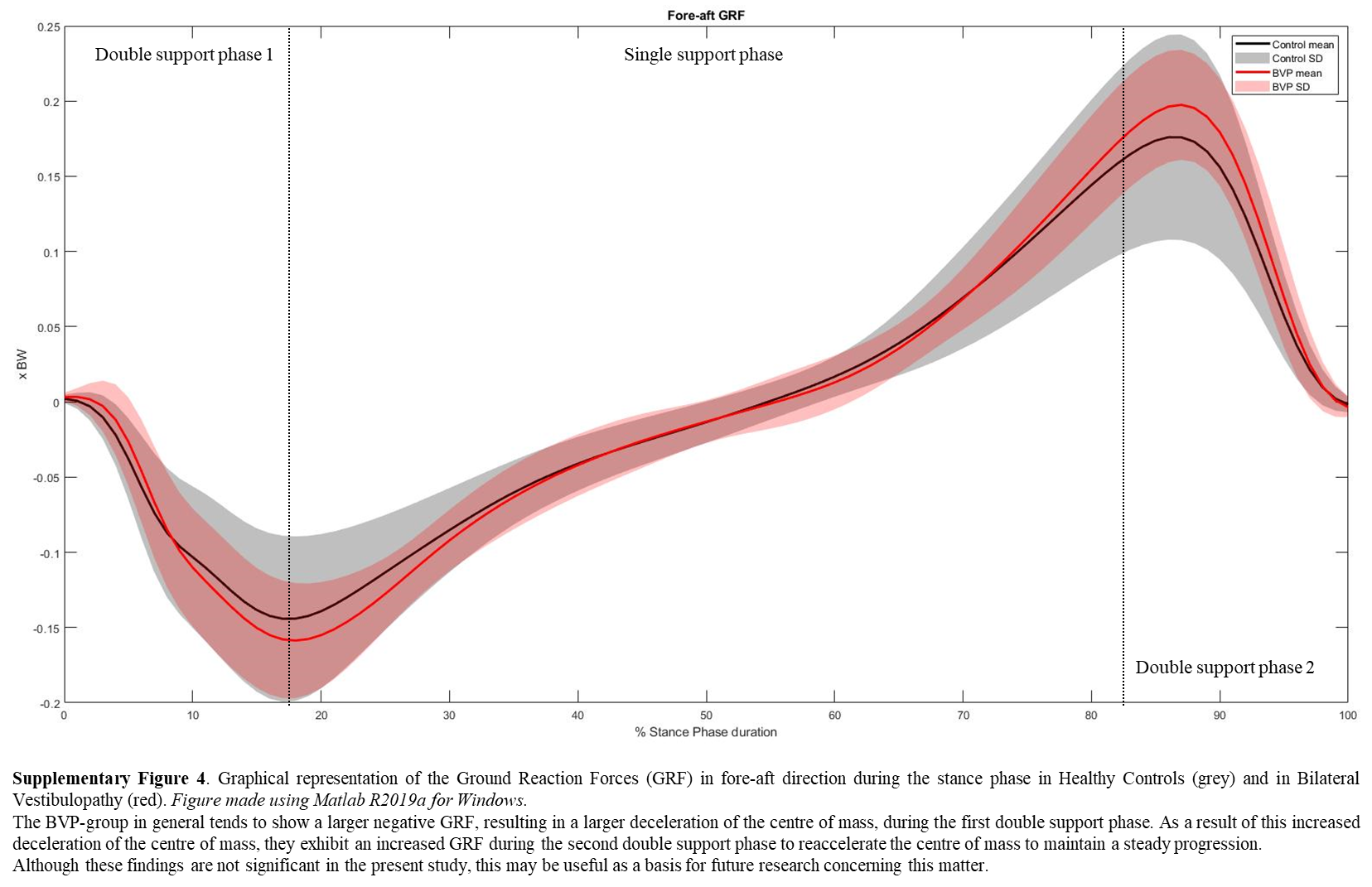


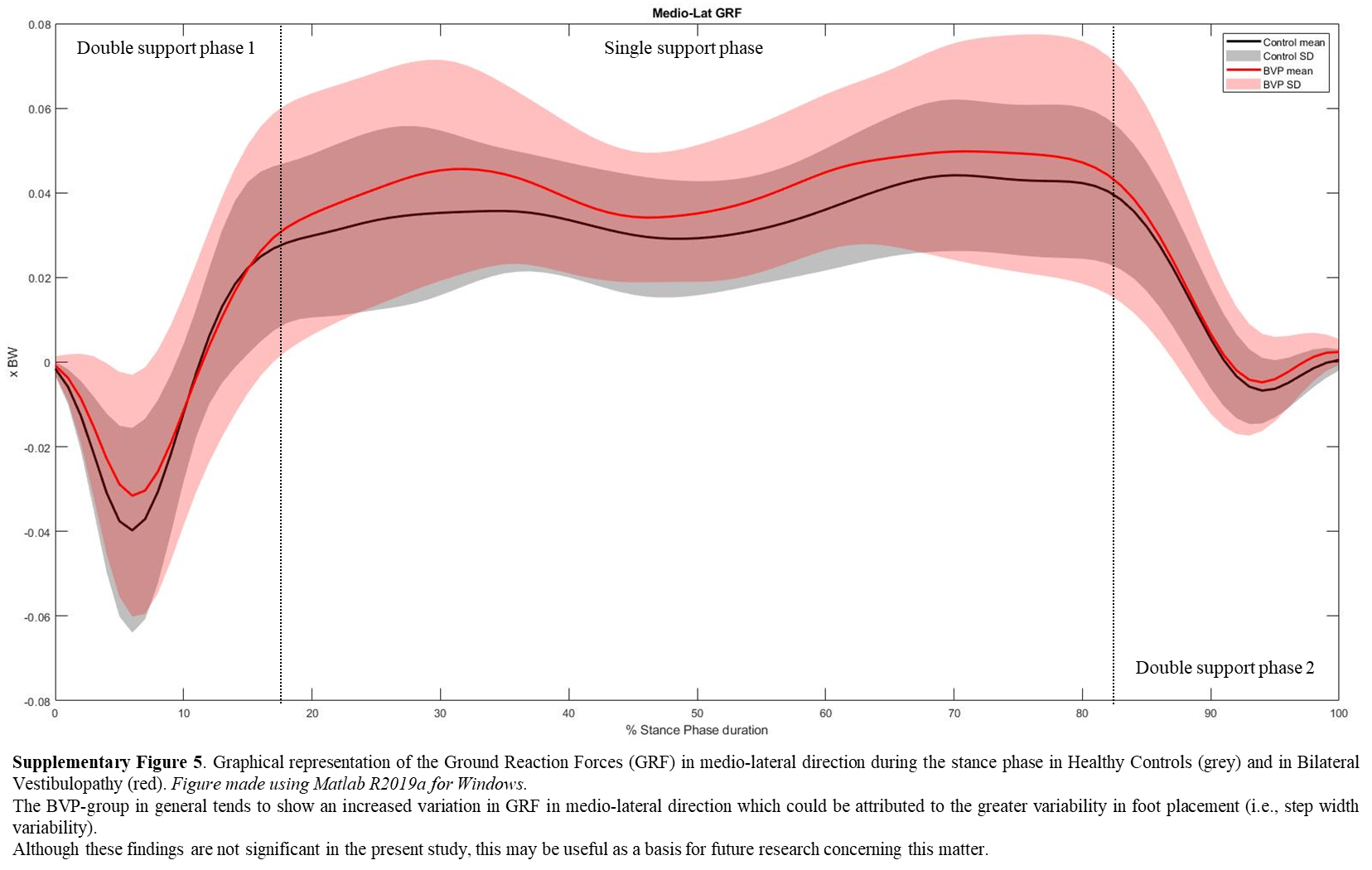


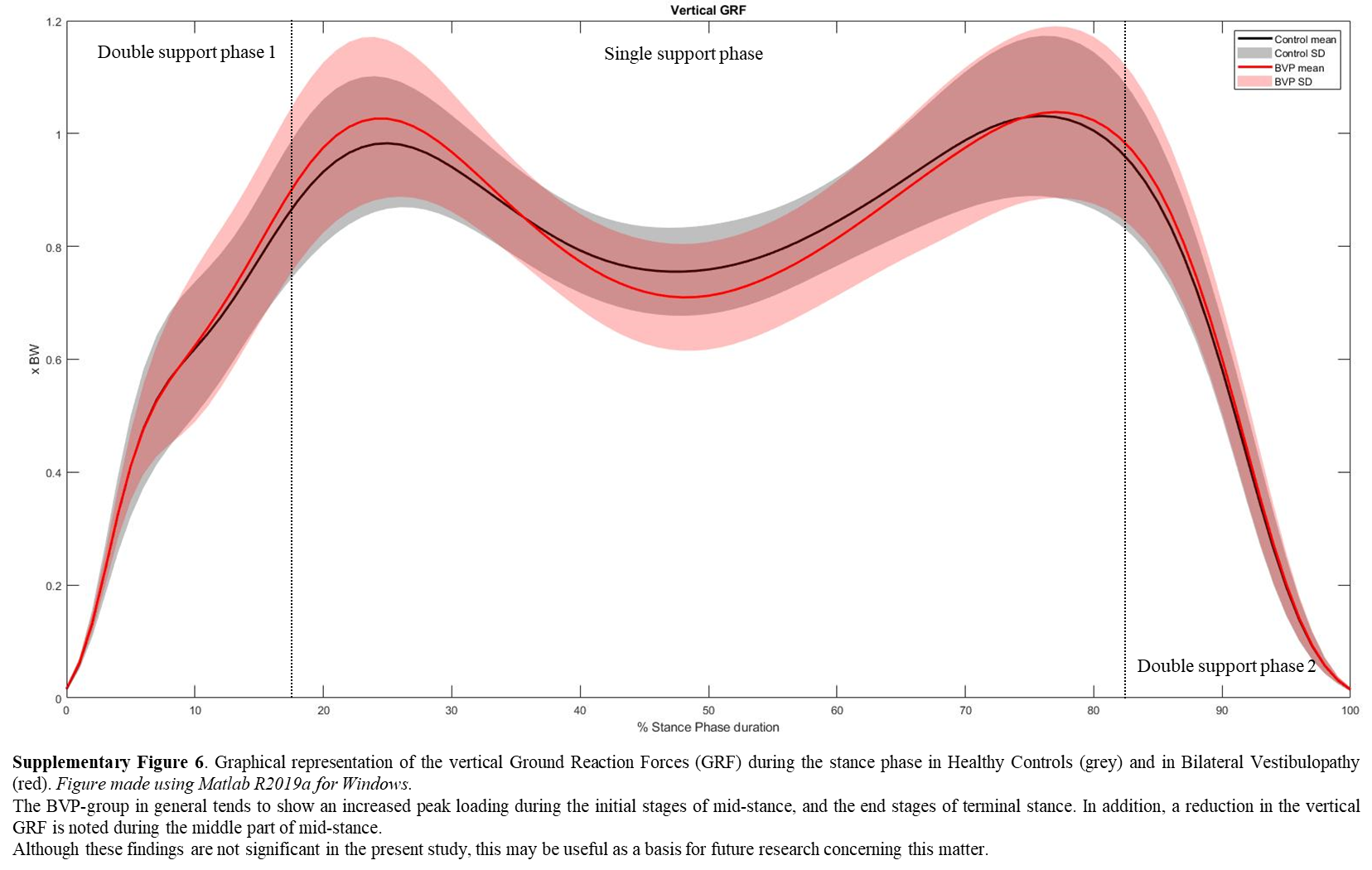


| **Supplementary Table 1**. Patient characteristics with vestibulo-ocolar reflex function testing as documented by the diagnostic criteria for bilateral vestibulopathy. | | | | | | | | | | |
| --- | --- | --- | --- | --- | --- | --- | --- | --- | --- | --- |
| **Subject** | **Sex** | **Age (years)** | **Time since onset (years)** | **Etiology** | | **Video Head Impulse Testing (gain)** | | **Caloric Testing (deg/s)** | | **Rotatory Chair Testing (gain)** |
|  |  |  |  | Left | Right | Left lateral SCC | Right lateral SCC | Left | Right |  |
| BVP01 | Female | 56.24 | 17 | Menière’s Disease | Idiopathic | 0.14 | 0.16 | 0 | 0 | 0.01 |
| BVP02 | Female | 74.05 | 5 | Idiopathic | | 0.74 | 1.01 | 4 | 3 | 0.1 |
| BVP03 | Male | 65.37 | 21 | Meningitis^†^ | | 0.45 | 0.56 | 4 | 5 | 0.1 |
| BVP04 | Male | 59.72 | 20 | Unknown | | 0.29 | 0.31 | 3 | 2 | 0.01 |
| BVP05 | Male | 72.19 | 4 | Idiopathic | | 0.27 | 0.40 | 0 | 3 | 0.05 |
| BVP06 | Female | 58.09 | 4 | Meningitis^†^ | | 0.85 | 0.98 | 0 | 1 | 0.15 |
| BVP07 | Male | 42.83 | 4 | Head trauma | | 0.88 | 1.06 | 11 | 19 | 0.05 |
| BVP08 | Male | 71.95 | 3 | Gentamicin^†^ | | 0.32 | 0.24 | 8 | 0 | 0.03 |
| BVP09 | Female | 70.71 | 6 | Idiopathic | | n.a. | n.a. | 0 | 0 | 0.02 |
| BVP10 | Male | 62.66 | 10 | Head trauma | | 0.12 | 0.04 | 0 | 0 | 0.04 |
| BVP11 | Male | 56.51 | 3 | Idiopathic | | 0.14 | 0.33 | 0 | 0 | 0.03 |
| BVP12 | Male | 62.92 | 6 | Idiopathic | Menière’s Disease | 0.70 | 0.39 | 4 | 0 | 0.30 |
| BVP13 | Male | 58.44 | 6 | Idiopathic | | 0.26 | 0.15 | 2 | 0 | 0.02 |
| BVP14 | Male | 55.65 | 7 | Idiopathic | Resection vestibular schwannoma | 0.84 | 0.38 | 0 | 0 | 0.19 |
| BVP15 | Male | 50.53 | 14 | Idiopathic | | 0.49 | 0.92 | 0 | 7 | 0.07 |
| BVP16 | Male | 33.37 | 30 | Meningitis | | 0.56 | 0.07 | 0 | 0 | 0.06 |
| BVP17 | Male | 49.00 | 6 | Genetic^†^ | | n.a. | n.a. | 0 | 0 | 0.40 |
| BVP18 | Male | 56.27 | 1 | Meningitis | | 0.69 | 0.42 | 0 | 0 | 0.20 |
| BVP19 | Female | 46.23 | 2 | DFNA9 | | 0.89 | 0.78 | 0 | 0 | 0.05 |
| BVP20 | Female | 63.31 | 20 | DFNA9 | | 0.62 | 0.61 | 0 | 0 | 0.05 |
| Note. SCC: Semi-circular canal; deg/s: degrees per second; A bilaterally reduced or absent angular VOR function has to be documented by: video Head Impulse Testing with a bilaterally pathological horizontal VOR gain <0.6 AND/OR a reduced caloric response (sum of bithermal maximal peak slow phase velocity on each side <6 deg/sec) AND/OR a reduced horizontal angular VOR gain <0.1 upon sinusoidal stimulation on a rotatory chair; †: probable etiology. | | | | | | | | | | |
